# Enzymatic Cross-Linking of Alkali Extracted Arabinoxylans: Gel Rheological and Structural Characteristics

**DOI:** 10.3390/ijms12095853

**Published:** 2011-09-09

**Authors:** Claudia M. Berlanga-Reyes, Elizabeth Carvajal-Millan, Jaime Lizardi-Mendoza, Alma R. Islas-Rubio, Agustin Rascón-Chu

**Affiliations:** 1 Centro de Investigación en Alimentación y Desarrollo, A.C. CTAOA, Biopolymers, Carretera a la Victoria Km 0.6, Hermosillo, Apdo. Postal 1735, Sonora, México; E-Mails: claudiaberlanga@hotmail.com (C.M.B.-R.); jalim@ciad.mx (J.L.-M.); 2 Centro de Investigación en Alimentación y Desarrollo, A.C. CTAOV, Carretera a la Victoria Km 0.6, Hermosillo, Apdo. Postal 1735, Sonora, México; E-Mails: aislas@ciad.mx (A.R.I-R.); arascon@ciad.mx (A.R.-C.)

**Keywords:** arabinoxylans, ferulic acid, laccase, gelation

## Abstract

Ferulated arabinoxylans were alkali-extracted from wheat bran at different incubation times (0.0, 0.5, 1.0, 1.5 and 2.0 h). Wheat bran ferulated arabinoxylans (WBAX) arabinose-to-xylose ratio, ferulic acid content, intrinsic viscosity and viscosimetric molecular weight values decreased as the incubation time of extraction increased. WBAX enzymatic cross-linking capability was affected by incubation time while an increase in WBAX concentration from 5 to 6% (w/v) favored gelation. The WBAX gels formed presented a macroporous structure with mesh size ranging from 40 to 119 nm and hardness values varying from 1.7 to 5 N.

## 1. Introduction

Wheat bran is a by-product of the commercial wheat milling process, which could be a potential source of added-value biomolecules such as hydrocolloids. Wheat bran contains fiber (approximately 58.4%, where 21.4% corresponds to arabinoxylans) [1]. The major part of the arabinoxylans portion in wheat bran is water-unextractable [2] and can be isolated by alkali extraction [3,4]. The extractability of these polysaccharides is based on their physical interactions and the covalent ester bonds between ferulic acid (FA) and other components such as lignin [5].

Arabinoxylans (AX) are built up of pentose sugars, mostly arabinose and xylose residues, and are therefore often referred to as pentosans [6]. Arabinoxylans consist of a backbone chain of β-(1→4)-d-xylopyranosyl units to which α-l-arabinofuranosyl substituents are attached through O–2 and/or O–3 [7]. Some of the arabinose residues are ester linked on (O)–5 to FA being called ferulated arabinoxylans [8].

Once extracted, arabinoxylans form highly viscous solutions with gelling capability by covalent cross–linking through dimerization of FA substituents under oxidative conditions (e.g., use of enzymatic free radical generating agents as laccase and peroxidase/H_2_O_2_ system) [9,10]. Diferulic acids (di–FA) and triferulic acid (tri–FA) [11,12] have been identified as covalently cross-linked structures in laccase-gelled AX. Both, covalent bridges (di–FA, tri–FA) and physical interactions between arabinoxylans chains, have been reported to be involved in the gelation process and the final gel properties [11,12]. The arabinoxylans properties such as solubility, viscosity and gelling capability are closely related to their chemical structure, conformation, and molecular interaction. Arabinoxylans gels present interesting properties like neutral taste and odor, high water absorption capability and stability to pH or electrolyte susceptibility [13].

In this study, ferulated arabinoxylans from wheat bran by-product have been alkali extracted and characterized and their gelling capability and the gel rheological and structural characteristics have been investigated.

## 2. Results and Discussion

### 2.1. WBAX Extraction and Characterization

**Figure 1:**
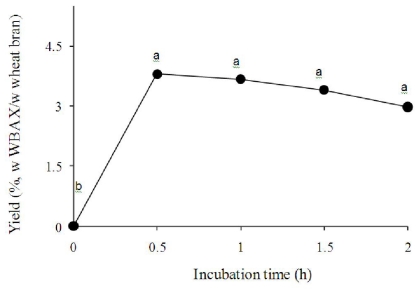
Yields of wheat bran ferulated arabinoxylans (WBAX) extracted under different incubation times in 0.5 M NaOH at 25 °C and 100 rpm in darkness. The presented results are averages of three replicates. Mean values with different letters are significantly different (*P* < 0.05).

In Figure 1, WBAX yields are shown as a function of extraction time. The yield of WBAX showed a statistically non–significant decrease as the time of extraction increased from 0.5 to 2 hours where yields values ranged from 3.8% to 3.0% (w WBAX/w wheat bran). By using calcium hydroxide 0.02 M (4 hours) and barium hydroxide 0.05 M (2 hours) at 20 °C a previous research [14] reported lower yield values (2% and 3%, respectively). The WBAX physic-chemical characteristics and enzymatic cross-linking capability were affected by the length of extraction time. Physico-chemical characteristics of the extracted WBAX are presented in Table 1. The ratio of arabinose-to-xylose (A/X) decreased from 0.83 to 0.76 as the time of extraction increased from 0.5 to 2.0 hours. The A/X ratios found in the present study indicate a highly branched structure, similar to that reported before [14–16] in wheat bran arabinoxylans. On the other hand, the FA content in WBAX was greatly affected by extraction time, registering values from 0.009 to 0.006 μg/mg WBAX. Higher FA contents (1–3 μg/mg) have been previously reported in wheat bran arabinoxylans extracted under mild conditions [15,16]. The intrinsic viscosity [η] and viscosimetric molecular weight (Mv) of WBAX registered a decrease from 206 to 184 mL/g and from 74 to 66 kDa, respectively, as the extraction time increased from 1.0 to 2.0 hours. [η] and Mv values obtained are in agreement with previous reports on ferulated arabinoxylans extracts of wheat bran [15,16]. [η] and Mv values could not be determined in WBAX extracted after 0.5 hours as this sample presented very low water solubility. In general, the effect of NaOH exposure on the yield and physico-chemical characteristics of WBAX can be attributed to the chemical hydrolysis of the different linkages in the arabinoxylan molecule (xylose-to-xylose, xylose-to-arabinose, arabinose-to-ferulic acid).

### 2.2. WBAX Enzymatic Cross-Linking and Gel Hardness

**Figure 2:**
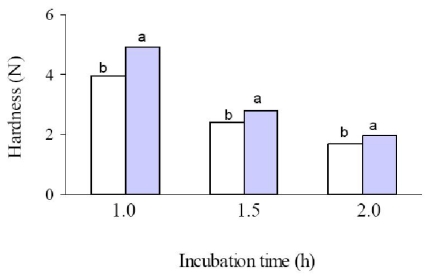
Hardness of WBAX gels at 5% (□) and 6% ( 
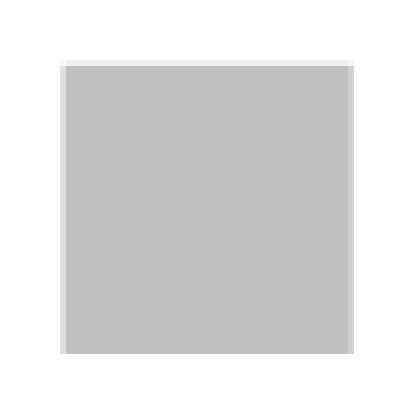
) in WBAX extracted using different incubation times. Values are means of triplicate measurements. All the given values are means of three determinations. Mean values for the same incubation time with different letters are significantly different (P < 0.05).

WBAX extracted with 1.0, 1.5 and 2.0 hours of NaOH exposure produced firm and brittle gels at 5 and 6% in WBAX (w/v) in the presence of laccase as a gelling agent. WBAX recovered after 0.5 hours of NaOH treatment did not form a homogenous solution; therefore gelation tests were not performed. The hardness of the WBAX gels obtained is presented in Figure 2. A significant increase in the gel hardness was registered when the polysaccharide concentration augmented from 5 to 6% (w/v) for WBAX extracted after 1.0, 1.5 or 2.0 hours NaOH exposure. Nevertheless, at the same polysaccharide concentration, increasing the WBAX extraction time decreased gel hardness by 57 and 60% for gels formed at 5 and 6% in WBAX (w/v), respectively. In general, WBAX gels hardness values (1–5 N) were higher than those previously reported for wheat flour and maize bran arabinoxylans (0.5–1 N) [12,17]. This decrease in gel hardness could be explained by the differences in the physico-chemical characteristics of the WBAX recovered as discussed above (Table 1). Arabinoxylans FA content and A/X ratio are related to gel hardness. A higher FA content could conduce to a higher content of cross-linking structures in the gel. The solubility of arabinoxylans is closely related to the presence of arabinose. The amount of arabinose affects the potential of arabinoxylan chains to interact with each other. A low A/X ratio (<0.4) may lead to precipitation of polysaccharide chains, affecting the gelling capability [13]. In the present study, WBAX recovered after 2 hours incubation presented A/X ratio, FA content, [η] and Mv values statistically significant lower than those of WBAX samples extracted after 1.0 and 1.5 hours of alkali treatment. In this context, the fact that increasing WBAX extraction time decreased gel hardness could be related to the reduction in the A/X ratio which decreases arabinoxylans solubility, to the lower FA content which could conduce to a low content of cross-linking structures and to the lower [η] and Mv values as a decrease in chain length could reduce the potential of the polymer chain to form intermolecular aggregates [13].

### 2.3. WBAX Gels Structure

The equilibrium swelling of the different WBAX gels at 5 and 6% (w/v) was reached between 8 and 10 h. The swelling ratio (q, g water/g WBAX) in WBAX gels increased with the length of the incubation time used for WBAX extraction (Table 2). This increase in q could be related to a decrease in the gel hardness (Figure 2). For all WBAX samples, an increase in WBAX concentration in the gel from 5 to 6% (w/v) reduced q values. The lower q values could be related to a more compact polymeric structure that limits water absorption [18,19]. The structural characteristics of WBAX gels (molecular weight between two cross-links, Mc; cross-linking density, ρc and mesh size, ξ) are presented in Table 2. As the extraction time of WBAX increased, higher Mc and ξ values of the gels formed were found while lower ρc were registered, which is consistent with the formation of weaker gels (Figure 2). When WBAX concentration in the gel was modified from 5 to 6% (w/v), Mc and ξ values decreased and ρc increased, confirming the formation of a stronger gel (Figure 2). Similar structural parameters have been reported for maize bran arabinoxylans gels [19] while in wheat flour arabinoxylans gels higher mesh sizes values (200–400 nm) were found [18]. The latter could be related to the differences in the structural characteristics of arabinoxylans from bran or endosperm cereal cells, such as molecular weight, ferulic acid content and A/X ratio.

## 3. Experimental Section

### 3.1. Materials

Ferulated arabinoxylans were isolated from wheat bran by-product kindly provided by a commercial wheat milling industry in Northern Mexico (Molinos La Fama). Laccase (benzenediol: oxygen oxidoreductase, E.C.1.10.3.2) from *Trametes versicolor*, sodium hydroxide, citric acid, sodium phosphate dibasic, hydrochloric acid and sulfuric acid were purchased from Sigma Chemical Co. (St. Louis, MO, USA).

### 3.2. Methods

#### 3.2.1. WBAX Extraction

Wheat bran was milled to a 20-mesh particle size using a CyclotecTM 1093 Mill (FOSS, Sweden). Wheat bran (500 g) was suspended in ethanol (2,500 mL) over 12 h at 25 °C and 100 rpm to remove lipophilic components. The ethanol treated bran was then filtered and subjected to starch gelatinization and enzyme inactivation (boiling for 30 min in 3500 mL of water). After boiling, wheat bran was recovered by filtration and treated with 2,500 mL of 0.5 N NaOH solution at 25 °C in darkness for 0, 0.5, 1.0, 1.5 or 2.0 hours with shaking (100 rpm). Residual bran was eliminated by filtration and the filtrate was centrifuged (12,096 g, 20 °C, 15 min). Supernatant was acidified to pH 4 with 3N HCl and then centrifuged (12,096 g, 20 °C, 15 min). Supernatant was then recovered and precipitated in 65% (v/v) ethanol for 4 h at 4 °C. Precipitate was recovered and dried by solvent exchange (80% (v/v) ethanol, absolute ethanol and acetone) to render wheat bran ferulated arabinoxylans (WBAX) powder.

#### 3.2.2. Laccase Activity

Laccase activity was measured at 25 °C from a laccase solution at 0.125 mg/mL in 0.1 M citrate-phosphate buffer pH 5.5 [10,12]. Syringaldazine 0.0216 mM in methanol was used as substrate. The enzymatic reaction was followed for 4 min at 530 nm.

#### 3.2.3. Sugar Composition

Sugar composition was determined after WBAX hydrolysis with 2 N trifluoroacetic acid at 120 °C for 2 h. The reaction was stopped on ice, the extract was evaporated under air at 40 °C and rinsed twice with water (200 μL) and resuspended in water (500 μL). All samples were filtered through 0.45 μm (Whatman) and analyzed by high performance liquid chromatography (HPLC) using a Supelcogel Pb column (300 × 7.8 mm; Supelco, Inc., Bellefont, PA, USA) eluted with 5 mM H_2_SO_4_ (filtered 0.2 μm, Whatman) at 0.6 mL/min and 50 °C [17]. A Varian 9012 HPLC with Varian 9,040 refractive index detector (Varian, St. Helens, Australia) and a Star Chromatography Workstation system control version 5.50 were used. Series of sugar calibration standards were prepared in HPLC grade water at appropriate concentrations for creating a calibration curve for each sugar of interest (xylose, arabinose, galactose, mannose and glucose) in the range of 0.2–12.0 mg/mL. Inositol was used as internal standard.

#### 3.2.4. Ferulic Acid

FA was quantified by reverse phase high-performance liquid chromatography (RP-HPLC) after a deesterification step, as described elsewhere [11]. An Alltima (Alltech, Deerfield, IL, USA) C18 5 μm column (250 × 4.6 mm) was used. Detection was by UV absorbance at 320 nm. Gradient elution was performed using acetonitrile and 0.05 M sodium acetate buffer, pH 4.0, at 1 mL/ min at 35 °C, in linear gradients from 15:85 to 35:65 in 30 min, from 35:65 to 60:40 in 0.5 min, from 60:40 to 15:85 in 4.5 min, and finally maintained at 15:85 for 5 min. A Waters 996 (Millipore Co., Milford, MA, USA) photodiode array detector was used to record the ferulic acid spectrum.

#### 3.2.5. Intrinsic Viscosity and Viscosimetric Molecular Weight

Specific viscosity (ηsp) of WBAX solutions was measured by registering WBAX solutions flow time in an Ubbelohde capillary viscometer at 25 ± 0.1 °C, immersed in a temperature controlled water bath. WBAX solutions were filtered using 0.45 μm membrane filters before viscosity measurements. The intrinsic viscosity ([η]) was estimated from relative viscosity measurements (ηrel) of WBAX solutions by extrapolation of Kraemer and Mead and Fouss curves to “zero” concentration [20,21]. The viscosimetric molecular weight (Mv) was calculated from the Mark-Houwink relationship, Mv = ([*η*]/k)^1/α^.

#### 3.2.6. WBAX Enzymatic Cross-Linking

WBAX solutions at 5 and 6% (w/v) were prepared in 0.1 M citrate phosphate buffer pH 5.5. WBAX solutions were mixed with 50 μL of laccase (1.675 nkat/mg WBAX). Gels were allowed to form for 6 h at 25 °C.

#### 3.2.7. Rheology

The hardness of 5% and 6% (w/v) WBAX gels made in 6 mL glass flasks of 30 mm height and 25 mm internal diameter was analyzed with a TA.XT2 Texture Analyzer (Stable Micro Systems, Godalming, England). The gels were deformed by compression at a constant speed of 1.0 mm/s to a distance of 4 mm from the gel surface using a cylindrical plunger (diameter 15 mm). The maximum force obtained from the force *vs*. distance curve was recorded as a measure of gel hardness [17].

#### 3.2.8. Gel Structure

After laccase addition, 2 mL WBAX solutions were quickly transferred to a 5 mL tip-cut-off syringe (diameter 1.5 cm) and allowed to gel for 6 h at 25 °C. After gelation, the gels were removed from the syringes, placed in glass vials and weighed. The gels were allowed to swell in 20 mL of 0.02% (w/v) sodium azide solution to prevent microbial contamination. During 10 h the samples were blotted and weighed. After weighing, a new aliquot of sodium azide solution was added to the gels. Gels were maintained at 25 °C during the test. The equilibrium swelling was reached when the weight of the samples changed by no more than 3%. The swelling ratio (*q*) was calculated as:

$$q=(W_{\text{s}}-W_{\text{d}})/W_{\text{d}}$$

where *W*_s_ is the weight of swollen gels and *W*_d_ is the weight of WBAX in the gel.

From swelling measurements, the molecular weight between two cross-links (Mc), the cross-linking density (ρc) and the mesh size (ξ) values of the different WBAX gels were obtained as described before [18].

## 4. Conclusions

Wheat bran arabinoxylan gels with different rheological and structural characteristics can be obtained by modifying the polysaccharide structural features such as molecular weight, A/X ratio and ferulic acid content or by using different polysaccharide concentrations. The incubation time of extraction of wheat bran showed a direct effect on the structural features of arabinoxylans investigated in this study. These differences could be reflected in the functional properties of the arabinoxylans gels such as the water absorption capacity. Further research is undergoing in order to explore the controlled release properties of these arabinoxylans gels.

## Figures and Tables

**Table 1:** Physico–chemical characteristics of wheat bran ferulated arabinoxylans (WBAX) extracted wth different incubation time.

Incubation time (h)	A/X ratio	FA (μg/mg WBAX)	[η] mL/g	Mv (kDa)
0.5	0.83 a	0.009 a	–	–
1.0	0.80 a	0.008 a	206 a	74 a
1.5	0.79 a	0.008 a	194 a	70 a
2.0	0.76 b	0.006 b	184 b	66 b

All values are means of three determinations. Mean values in the same column with different letters are significantly different (P < 0.05).

**Table 2:** Structural characteristics of WBAX gels formed at different polysaccharide concentrations and with distinct incubation times during WBAX extraction.

	WBAX (% w/v)							
	5.0				6.0			
Incubation time (h)	q (g water/g WBAX)	Mc × 10^3^ (g/mol)	ρ_c_ × 10^−6^ (mol/cm^3^)	ξ (nm)	q (g water/g WBAX)	Mc × 10^3^ (g/mol)	ρ_c_ × 10^−6^ (mol/cm^3^)	ξ (nm)
		
1.0	8.6 b	29 c	59 a	57 c	6.6 b	16 b	102 a	40 b
1.5	13.4 a	40 b	41 b	72 b	11.6 a	19 a	84 b	44 a
2.0	11.2 a	80 a	21 c	119 a	10.3 a	18 a	95 b	41 b

All the given values are means of three determinations. Mean values in the same column with different letters are significantly different (*P* < 0.05).
